# Methylsulfonylmethane (MSM) Mitigates Cisplatin-Induced Early Oxidative Testicular Dysfunction Through Modulation of Antioxidant Defense and GPX4 Response

**DOI:** 10.3390/nu18142392

**Published:** 2026-07-22

**Authors:** Pelin İsmailoğlu, Zehra Topal Suzan, Esra Deniz, Adnan Yılmaz, Sibel Mataracı Karakaş, Nevnihal Akbaytürk, Hatice Sevim Nalkıran, İhsan Nalkıran, Ünzile Yaman, Şenay Çakıroğlu, Levent Tümkaya

**Affiliations:** 1 Department of Anatomy, Faculty of Medicine, Recep Tayyip Erdoğan University, Rize 53020, Turkey; 2 Department of Histology and Embryology, Faculty of Medicine, Recep Tayyip Erdoğan University, Rize 53020, Turkey; zehra.suzan@erdogan.edu.tr; 3 Department of Pharmacology, Faculty of Medicine, Recep Tayyip Erdoğan University, Rize 53020, Turkey; esra.deniz@erdogan.edu.tr; 4 Department of Biochemistry, Faculty of Medicine, Recep Tayyip Erdoğan University, Rize 53020, Turkey; adnan.yilmaz@erdogan.edu.tr (A.Y.); sibel.karakas@erdogan.edu.tr (S.M.K.); 5 Department of Anatomy, Faculty of Medicine, Giresun University, Giresun 28100, Turkey; nevnihal.akbayturk@giresun.edu.tr; 6 Department of Medical Biology, Faculty of Medicine, Recep Tayyip Erdoğan University, Rize 53020, Turkey; hatice.sevim@erdogan.edu.tr (H.S.N.); ihsan.nalkiran@erdogan.edu.tr (İ.N.); 7 Department of Pharmaceutical Toxicology, Faculty of Pharmacy, Izmir Katip Celebi University, Izmir 35620, Turkey; unzile.yaman@ikcu.edu.tr; 8 Experimental Animals Application and Research Center, Recep Tayyip Erdoğan University, Rize 53020, Turkey; senay.cakiroglu@erdogan.edu.tr; 9 Department of Histology and Embryology, Faculty of Medicine, Ondokuz Mayıs University, Samsun 55200, Turkey; levent.tumkaya@omu.edu.tr

**Keywords:** cisplatin, oxidative stress, methylsulfonylmethane, GPX4, antioxidant defense, testosterone, testicular toxicity, redox homeostasis

## Abstract

**Background/Objectives**: Cisplatin-induced testicular toxicity is commonly associated with oxidative stress; however, the early biological events preceding overt tissue injury remain incompletely characterized. Identifying interventions capable of preserving redox homeostasis during this subacute phase may improve our understanding of the initial mechanisms underlying testicular dysfunction. This study investigated whether methylsulfonylmethane (MSM), a naturally occurring organosulfur compound with antioxidant properties, modulates early oxidative responses in a subacute rat model of cisplatin-induced testicular toxicity. **Methods**: Thirty-two adult male Sprague–Dawley rats were randomly assigned to Control, Cisplatin (CIS), MSM, and CIS + MSM groups (*n* = 8/group). MSM (500 mg/kg/day, intraperitoneally) was administered for 10 consecutive days, while a single dose of cisplatin (7 mg/kg, intraperitoneally) was given on day 7. Oxidative stress biomarkers, antioxidant enzyme activities, intratesticular testosterone concentrations, inflammatory cytokines, GPX4 and HO-1 protein expression, histopathological alterations, and correlation analyses were evaluated. **Results**: Cisplatin exposure induced a marked oxidative imbalance, evidenced by increased malondialdehyde levels and reduced superoxide dismutase, catalase, glutathione peroxidase, and intratesticular testosterone concentrations (*p* < 0.05). MSM administration attenuated lipid peroxidation, restored antioxidant enzyme activities, and preserved intratesticular testosterone levels. Western blot analysis demonstrated a significant increase in GPX4 protein expression following cisplatin exposure, whereas MSM normalized GPX4 expression toward control values. In contrast, HO-1 expression and intratesticular IL-6 and TNF-α levels did not differ among the experimental groups. Histopathological evaluation revealed only mild structural alterations without corresponding differences in Johnsen score, indicating that biochemical and molecular disturbances preceded overt tissue degeneration. Correlation analysis further demonstrated close associations between antioxidant defense and preservation of endocrine function. **Conclusions**: Subacute cisplatin exposure primarily disrupted testicular redox homeostasis before prominent histopathological injury became evident. MSM was associated with attenuation of these early oxidative alterations accompanied by improved endogenous antioxidant enzyme activity, higher intratesticular testosterone concentrations, and normalization of GPX4 expression. These findings suggest that MSM may contribute to the maintenance of testicular redox homeostasis during the early phase of cisplatin-induced toxicity under the present experimental conditions.

## 1. Introduction

Cisplatin is a platinum based chemotherapeutic agent used in the treatment of several solid malignancies, including ovarian, bladder, lung, testicular, and head and neck cancers [1]. Despite its well established antitumor efficacy, cisplatin administration is frequently associated with adverse effects involving several non-target organs, including the male reproductive system [2]. Testicular toxicity represents an important clinical concern because it may impair steroidogenesis, spermatogenesis, and ultimately male reproductive function, particularly in young cancer survivors [1,3]. Accordingly, understanding the early biological alterations following cisplatin exposure may provide further insight into the mechanisms underlying cisplatin-induced testicular injury [4]. Accumulating evidence indicates that oxidative stress is considered one of the principal mechanisms to cisplatin induced testicular damage [5]. Excessive production of reactive oxygen species (ROS) following cisplatin exposure disrupts intracellular redox homeostasis, leading to lipid peroxidation, mitochondrial dysfunction, oxidation of cellular macromolecules, and impairment of endogenous antioxidant defense systems [6]. Because the testis exhibits high metabolic activity, continuous germ cell turnover, and relatively limited antioxidant capacity, it is particularly susceptible to oxidative injury. Under physiological conditions, antioxidant enzymes, including superoxide dismutase (SOD), catalase (CAT), and glutathione peroxidase (GPx), play essential roles in maintaining cellular redox balance [5,6]. Disruption of these antioxidant defense mechanisms together with increased lipid peroxidation may impair Leydig cell function and contribute to reduced testosterone production [3]. Recent experimental studies further suggest that these biochemical alterations may become evident before overt histopathological degeneration develops, indicating that early oxidative responses may represent the initial stage of cisplatin-induced testicular dysfunction [1,2].

In addition to conventional oxidative stress markers, increasing attention has focused on endogenous adaptive defense mechanisms activated during oxidative injury [7]. Among these, glutathione peroxidase-4 (GPX4) is recognized as an essential antioxidant enzyme that protects membrane phospholipids against oxidative damage by reducing phospholipid hydroperoxides [8,9]. Heme oxygenase-1 (HO-1), another stress-inducible cytoprotective protein, also contributes to the maintenance of cellular redox homeostasis under conditions of oxidative stress [10]. Rather than representing irreversible tissue injury, alterations in the expression of these proteins may reflect early adaptive cellular responses aimed at maintaining redox balance during the initial phase of oxidative stress [6]. Accordingly, evaluation of GPX4 and HO-1 together with biochemical oxidative stress markers may provide additional insight into the molecular events accompanying early cisplatin-induced testicular injury [8,10,11].

MSM is a naturally occurring organosulfur compound that has attracted considerable interest because of its antioxidant and anti-inflammatory properties [12,13]. Experimental studies have demonstrated that MSM reduces oxidative damage by scavenging reactive oxygen species, enhancing endogenous antioxidant enzyme activity, and improving cellular redox homeostasis in various experimental models [14]. In addition, MSM has been reported to modulate inflammatory signaling pathways and preserve tissue integrity under conditions associated with oxidative stress [14]. However, despite increasing interest in the use of nutraceuticals as supportive approaches during chemotherapy, the potential effects of MSM on the early oxidative phase of cisplatin-induced testicular dysfunction remain insufficiently characterized, particularly with respect to adaptive antioxidant responses involving GPX4 [8,12,13,14].

Most experimental studies investigating protective strategies against cisplatin-induced testicular toxicity have focused on advanced stages of tissue injury characterized by pronounced histopathological degeneration and impaired spermatogenesis [3,4,7]. In contrast, comparatively little attention has been given to the early biochemical and molecular alterations that occur before overt structural damage becomes evident. Previous experimental studies suggest that these early biochemical and molecular changes may be detectable before overt histopathological injury develops, supporting the use of an early subacute experimental model [4,15]. A better understanding of this subacute phase may improve our understanding of the temporal progression of cisplatin-induced testicular toxicity and provide insight into the early events underlying testicular dysfunction. Although the antioxidant properties of MSM have been demonstrated in various experimental models, its potential role during the early phase of cisplatin-induced testicular toxicity remains incompletely understood [13,16]. Therefore, the present study was designed to investigate whether (MSM) exerts measurable effects during the early (10 day) subacute phase of cisplatin-induced testicular toxicity. Specifically, we sought to determine whether MSM may influence the early oxidative and molecular responses associated with cisplatin exposure before marked histopathological alterations become apparent. To address this question, oxidative stress biomarkers, endogenous antioxidant defense, intratesticular testosterone levels, GPX4 and HO-1 protein expression, inflammatory cytokines, and histomorphological changes were evaluated. Given its reported antioxidant properties of MSM, we hypothesized that MSM may influence the early biological responses to cisplatin exposure by supporting redox homeostasis and endogenous antioxidant defense during the subacute phase of testicular toxicity.

## 2. Materials and Methods

### 2.1. Chemicals and Reagents

Cisplatin (Cipintu®; Doğa İlaç, Ankara, Turkey) was obtained from a commercial source (Koçak Farma, Istanbul, Turkey). Methylsulfonylmethane (MSM) was purchased commercially and freshly dissolved in sterile physiological saline (0.9% NaCl) (Osel İlaç, Ankara, Turkey) immediately before administration. Ketamine (Keta-Control (Doğa İlaç, Ankara, Turkey) and xylazine (Doğalazin (Doğa İlaç, Ankara, Turkey) were used for anesthesia and euthanasia procedures. Unless otherwise specified, all chemicals and reagents used in this study were of analytical grade.

### 2.2. Animals and Experimental Protocol

This experimental study included thirty-two adult male Sprague–Dawley rats obtained from the Recep Tayyip Erdoğan University Experimental Animals Application and Research Center (Rize, Turkey). At the beginning of the study, the animals were 16–17 weeks old and weighed 400–450 g. All experimental procedures were approved by the Recep Tayyip Erdoğan University Animal Experiments Local Ethics Committee (Approval date: 27 June 2025; Decision No. 2025/31) and were conducted in accordance with the National Institutes of Health Guide for the Care and Use of Laboratory Animals and the ARRIVE guidelines.

Rats were housed in standard polypropylene cages under controlled environmental conditions: 22 ± 2 °C temperature, 45–65% relative humidity, and a 12 h light/dark cycle. The facility was equipped with a HEPA-filtered ventilation system. Animals had ad libitum access to standard laboratory chow and water. Cage bedding consisted of sterile, dust-free wood shavings which were changed regularly to maintain hygienic conditions. Every effort was made to minimize animal stress and ensure animal welfare throughout the experimental period. After one week of acclimatization, the rats were randomly divided into four groups (*n* = 8 per group). MSM was freshly prepared daily by dissolving it in physiological saline, which was then administered according to the body weight of each animal. Throughout the experimental period, the animals’ general health status and body weight changes were monitored daily.

Control group: Received daily intraperitoneal (i.p.) injections of physiological saline for 10 days.

Cisplatin (CIS) group: Received a single i.p. injection of 7 mg/kg cisplatin on day 7.

MSM group: Received MSM (500 mg/kg/day, i.p.) for 10 consecutive days.

CIS + MSM group: Received MSM (500 mg/kg/day, i.p.) for 10 days, with a single i.p. dose of 7 mg/kg cisplatin on day 7.

Animals were euthanized on day 10 to evaluate biochemical, molecular, and histomorphological alterations occurring during the subacute phase of cisplatin-induced testicular toxicity.

### 2.3. Tissue Collection

Animals were anesthetized with ketamine (80 mg/kg) and xylazine (10 mg/kg) prior to sacrifice. Blood samples were collected by intracardiac puncture and centrifuged to obtain serum. Both testes were immediately excised and rinsed with ice-cold physiological saline. One testis from each animal was fixed in 10% neutral buffered formalin for histopathological and immunohistochemical analyses, whereas the contralateral testis was snap-frozen in liquid nitrogen and stored at −80 °C until biochemical and Western blot analyses. Two animals were unavailable for tissue collection: one animal in the Control group died during the experimental period, and one animal in the CIS + MSM group was found dead in its cage on the final experimental day before the scheduled tissue collection.

### 2.4. Biochemical Analyses

Following euthanasia, blood samples were collected by intracardiac puncture, centrifuged at 3500× *g* rpm for 15 min, and the obtained serum aliquots were stored at −80 °C until analysis. Both testes were rapidly excised, rinsed with ice-cold physiological saline to remove residual blood, blotted dry, and stored at −80 °C until biochemical analyses. Frozen testicular tissues were homogenized in ice-cold phosphate buffer (20 mM sodium phosphate containing 140 mM of potassium chloride, pH 7.4) using a TissueLyser II homogenizer (Qiagen, Hilden, Germany) at a tissue-to-buffer ratio of 1:9 (*w*/*v*). The homogenates were centrifuged at 800× *g* for 10 min at 4 °C, and the resulting supernatants were collected for subsequent biochemical analyses.

Lipid peroxidation was assessed by measuring malondialdehyde (MDA) using the thiobarbituric acid reactive substances (TBARS) assay according to the modified method of Ohkawa et al., whereas reduced glutathione (GSH) levels were determined using Ellman’s colorimetric method. To comprehensively evaluate the early biochemical responses to cisplatin exposure, testicular concentrations of superoxide dismutase (SOD), glutathione peroxidase (GPX), catalase (CAT), testosterone, interleukin-6 (IL-6), and tumor necrosis factor-α (TNF-α) were measured using commercially available sandwich enzyme-linked immunosorbent assay (ELISA) kits (YL Biont, Shanghai, China) according to the manufacturers’ protocols. Optical densities were measured at 450 nm, and analyte concentrations were calculated from standard calibration curves. Results were expressed as ng/g tissue, nmol/g tissue, mmol/g tissue, or mIU/g tissue, as appropriate.

### 2.5. Western Blot Analysis

Total protein was extracted from frozen testicular tissue samples using RIPA lysis buffer supplemented with a protease inhibitor cocktail. Briefly, approximately 30 mg of tissue from each sample was homogenized in ice-cold lysis buffer and centrifuged at 13,000× *g* for 10 min at 4 °C. The resulting supernatants were collected, and protein concentrations were determined using the Pierce Dilution-Free Rapid Gold BCA Protein Assay Kit (Thermo Fisher Scientific, Waltham, MA, USA; Cat. No. A55861).

Equal amounts of protein (20–30 μg) were mixed with sample loading buffer, denatured at 95–99 °C for 5 min, separated by 10% SDS-PAGE, and transferred onto polyvinylidene difluoride (PVDF) membranes (GVS North America, Wilmington, NC, USA; Cat. No. 1212639). Membranes were blocked with 5% non-fat dry milk prepared in Tris-buffered saline containing 0.1% Tween-20 (TBST) for 1 h at room temperature and subsequently incubated overnight at 4 °C with primary antibodies against GPX4 (Proteintech, Rosemont, IL, USA; Cat. No. 67763-1-Ig), HO-1 (Cell Signaling Technology, Danvers, MA, USA; Cat. No. 43966; 1:1000), and β-tubulin (Cell Signaling Technology, Danvers, MA, USA; Cat. No. 2146S; 1:1000), which served as the loading control.

Following washing with TBST, membranes were incubated with horseradish peroxidase (HRP)-conjugated secondary antibodies, including anti-mouse IgG (Cell Signaling Technology, Danvers, MA, USA; Cat. No. 7076S; 1:2000) and goat anti-rabbit IgG H&L (Abcam, Cambridge, UK; Cat. No. ab205718; 1:1000), for 1 h at room temperature. Immunoreactive bands were visualized using Clarity Western ECL Substrate (Bio-Rad Laboratories, Hercules, CA, USA; Cat. No. 1705060) and detected with a ChemiDoc Imaging System (Bio-Rad Laboratories, Hercules, CA, USA).

Band intensities were quantified using ImageJ software (version 1.53t; National Institutes of Health, Bethesda, MD, USA), and target protein expression levels were normalized to β-tubulin. The expression levels of GPX4 and HO-1 were evaluated as indicators of oxidative stress–associated antioxidant responses in testicular tissue. All Western blot experiments were independently repeated three times to confirm reproducibility.

### 2.6. Histopathological and Immunohistochemical Evaluation

Testicular tissues were fixed in 10% neutral buffered formalin, routinely processed, embedded in paraffin, and sectioned at 4–5 μm thickness. Sections were stained with hematoxylin and eosin (H&E) for histopathological evaluation. Spermatogenesis was semi-quantitatively assessed using the Johnsen Testicular Biopsy Score (JTBS), in which seminiferous tubules were scored from 1 to 10 according to the degree of spermatogenic maturation. For immunohistochemical analysis, sections were processed using an automated staining system (Bond-Max, Leica Biosystems, Wetzlar, Germany). Representative H&E images were captured using an Olympus BX51 microscope equipped with a DP74 digital camera.

### 2.7. Statistical Analysis

Statistical analyses were performed using SPSS version 18.0 (IBM Corp., Armonk, NY, USA). Data distribution was assessed using the Shapiro–Wilk test. Parametric variables are presented as mean ± standard deviation (SD) and were analyzed using one-way analysis of variance (ANOVA) followed by the least significant difference (LSD) post hoc test. Variables that did not satisfy the assumptions of normality are presented as median (interquartile range, IQR) and were analyzed using the Kruskal–Wallis test followed by Mann–Whitney U tests for pairwise comparisons where appropriate. Histopathological and immunohistochemical scoring data were analyzed using non-parametric methods because of their semi-quantitative nature and the presence of missing observations resulting from technical tissue-processing procedures. Missing values were not imputed. A two-sided *p* value < 0.05 was considered statistically significant.

## 3. Results

### 3.1. MSM Attenuated Cisplatin-İnduced Oxidative Stress and Restored Antioxidant Defense in Testicular Tissue

To evaluate the effects of methylsulfonylmethane (MSM) on early oxidative stress during subacute cisplatin-induced testicular toxicity, lipid peroxidation and endogenous antioxidant defense markers were assessed in testicular tissue (Table 1).

Cisplatin disrupted testicular redox homeostasis. Testicular malondialdehyde (MDA) levels were significantly higher in the cisplatin group than in the control group (37 [36.1–38.7] vs. 31 [27.7–33.1] nmol/g tissue, *p* = 0.025). Co-administration of MSM reduced MDA levels compared with the cisplatin group, restoring values close to those observed in the control group (31 [29.9–34.2] nmol/g tissue, *p* < 0.05). In contrast, reduced glutathione (GSH) concentrations did not differ among the experimental groups (*p* = 0.434). Consistent with the increase in lipid peroxidation, cisplatin suppressed endogenous antioxidant defense enzymes. Superoxide dismutase (SOD), glutathione peroxidase (GPX), and catalase (CAT) levels were significantly lower in the cisplatin group than in the control group (*p* = 0.035, *p* = 0.006, and *p* = 0.003, respectively). MSM administration restored SOD and CAT levels compared with the cisplatin group, with values approaching those of the control animals. GPX levels also increased following MSM treatment compared with the cisplatin group, although they remained below control values. Taken together, these findings indicate that cisplatin induced an early oxidative imbalance characterized by increased lipid peroxidation and reduced antioxidant defense. MSM administration partially restored antioxidant enzyme levels, particularly SOD and CAT, while reducing lipid peroxidation during the subacute phase of cisplatin-induced testicular toxicity.

### 3.2. MSM Preserved İntratesticular Testosterone Levels Following Cisplatin Exposure

Since Leydig cell function is particularly susceptible to oxidative imbalance, intratesticular testosterone concentrations were subsequently evaluated (Table 2). Cisplatin administration resulted in reduction in intratesticular testosterone levels compared with the control group (0.21 [0.17–0.21] vs. 0.22 [0.21–0.23] mIU/g tissue, *p* < 0.05). Testosterone concentrations in the MSM group did not differ from those observed in the cisplatin group. In contrast, co-administration of MSM with cisplatin increased intratesticular testosterone levels compared with the cisplatin group and restored values to a level comparable with the control group. Furthermore, testosterone levels in the CIS + MSM group were significantly higher than those in the MSM-alone group. These findings suggest that MSM contributes to the preservation of intratesticular testosterone levels during the early phase of cisplatin-induced testicular toxicity (Table 2).

### 3.3. MSM Modulated GPX4-Associated Antioxidant Responses in Subacute Cisplatin-Induced Testicular Toxicity

**Figure 1:**
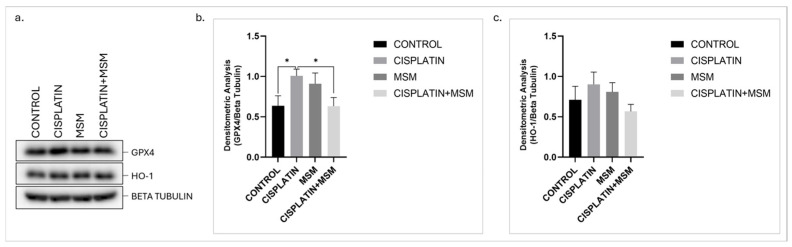
Effects of methylsulfonylmethane (MSM) on GPX4 and HO-1 protein expression in cisplatin-induced testicular toxicity. (**a**) Representative Western blot images showing GPX4 and HO-1 protein expression in the control, cisplatin, MSM, and cisplatin + MSM groups. β-Tubulin was used as the loading control. (**b**) Densitometric analysis of GPX4 protein expression normalized to β-tubulin. (**c**) Densitometric analysis of HO-1 protein expression normalized to β-tubulin. Data are presented as mean ± standard deviation (SD). Statistical comparisons between groups are indicated in the graphs (* *p* < 0.05). All Western blot experiments were independently repeated three times (*n* = 3) to confirm reproducibility.

To further characterize changes in antioxidant-related protein expression, GPX4 and HO-1 protein expression were evaluated by Western blot analysis (Figure 1a). Densitometric analysis showed a significant increase in GPX4 protein expression following cisplatin administration compared with the control group (Figure 1b). Although GPX4 expression was also elevated in the MSM-alone group, co-administration of MSM with cisplatin reduced GPX4 expression compared with the cisplatin group, resulting in values that approached those observed in the control group.

In contrast, HO-1 protein expression showed only modest variations among the experimental groups, and no statistically significant differences were detected (Figure 1c). Although HO-1 expression tended to increase following cisplatin administration and decrease in the CIS + MSM group, these differences did not reach statistical significance.

Overall, Western blot analysis showed a significant increase in GPX4 protein expression following cisplatin administration, whereas HO-1 protein expression did not differ significantly among the experimental groups. GPX4 protein expression was lower in the CIS + MSM group than in the cisplatin group and approached the levels observed in the control group, while no significant effect of MSM on HO-1 protein expression was observed.

### 3.4. Inflammatory Cytokines Remained Unchanged During the Subacute Phase

To determine whether early oxidative alterations were accompanied by an inflammatory response, intratesticular interleukin-6 (IL-6) and tumor necrosis factor-α (TNF-α) concentrations were evaluated (Table 3). No statistically significant differences were observed in IL-6 or TNF-α levels among the experimental groups (*p* = 0.114 and *p* = 0.253, respectively). Although both cytokines showed minor variations following cisplatin administration and MSM treatment, these changes did not reach statistical significance. These findings suggest that overt inflammatory cytokine responses were not evident during the subacute phase under the present experimental conditions.

### 3.5. Histopathological Alterations Remained Limited During the Subacute Phase

**Figure 2:**
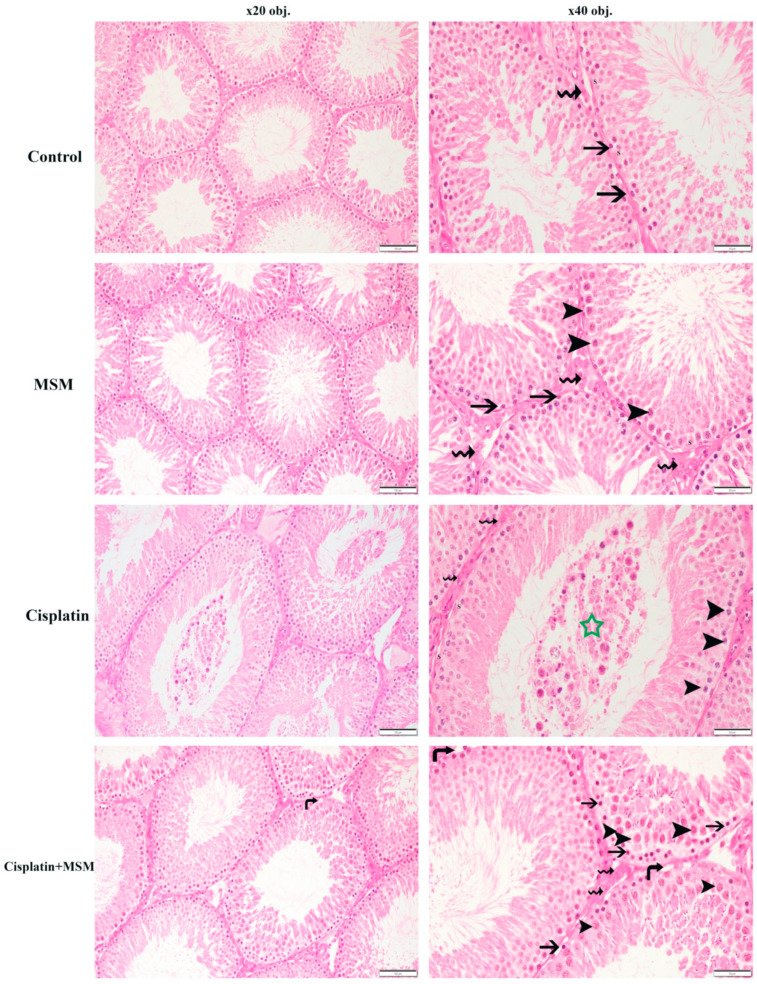
Representative photographs of testicular tissues stained with H&E. Arrow: spermatogonium; arrowhead: primary spermatogonium; curved arrow: Leydig cell; green star: debris of germ cells in the lumen; bent arrow: slight vacuolization; S: Sertoli cell.

Histopathological examination was performed to determine whether the biochemical and molecular alterations observed following cisplatin exposure were accompanied by overt structural damage in testicular tissue. Representative H&E-stained sections are shown in Figure 2, and quantitative Johnsen score analysis is summarized in Table 4.

The control and MSM groups exhibited preserved seminiferous tubular architecture with regular organization of the germinal epithelium, intact spermatogenic cell layers, and normal interstitial Leydig cells (Figure 2). In contrast, testicular sections from the cisplatin-treated group showed mild histomorphological alterations, including focal tubular deformation, limited sloughing of germ cells into the tubular lumen, and subtle epithelial irregularity. Nevertheless, the overall seminiferous tubular organization remained largely preserved. In animals receiving combined cisplatin and MSM treatment, seminiferous tubules generally retained normal architecture with preservation of the germinal epithelial layers. Although occasional vacuolization was observed in a limited number of tubules, no apparent disruption of spermatogenic organization was detected (Figure 2).

Consistent with these morphological observations, quantitative assessment demonstrated no statistically significant differences in Johnsen score among the experimental groups (H = 5.745, *p* = 0.125) (Table 4). Collectively, these findings indicate that although cisplatin induced mild histomorphological alterations during the short-term experimental period, overt seminiferous tubular degeneration had not yet developed. This observation is consistent with the biochemical and molecular findings, suggesting that biochemical and molecular alterations were detectable at the examined time point, whereas structural changes remained limited under the present experimental conditions.

### 3.6. Correlation Analysis Linked Antioxidant Defense with Testosterone Preservation

**Figure 3:**
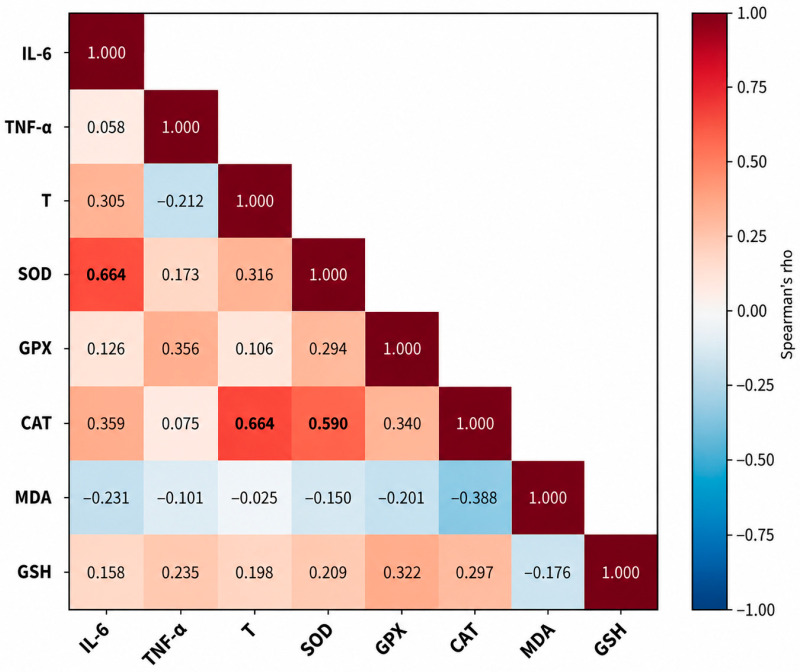
Heat map showing Spearman’s correlation coefficients among inflammatory, hormonal, and oxidative stress-related parameters in testicular tissue. Strong positive correlations were observed between IL-6 and SOD (ρ = 0.664), testosterone and CAT (ρ = 0.664), and SOD and CAT (ρ = 0.590). A negative correlation was observed between CAT and MDA (ρ = −0.388), suggesting that higher catalase levels may be associated with reduced lipid peroxidation. Overall, the correlation pattern indicates that antioxidant defense, particularly the SOD–CAT axis, is closely related to testosterone preservation and redox homeostasis in cisplatin-induced testicular toxicity. Overall, the observed correlations complement the biochemical findings but should be interpreted cautiously because correlation analyses cannot establish causality.

Spearman correlation analysis was performed to explore the relationships among oxidative stress biomarkers, antioxidant enzymes, inflammatory cytokines, and intratesticular testosterone levels (Figure 3).

## 4. Discussion

The principal finding of the present study is that subacute cisplatin exposure induced an early oxidative imbalance in testicular tissue, characterized by increased lipid peroxidation, impaired endogenous antioxidant defense, and reduced intratesticular testosterone levels before overt histopathological degeneration became evident. In contrast, intratesticular IL-6 and TNF-α levels remained unchanged at the examined time point. These findings indicate that the inflammatory markers evaluated in the present study were not significantly altered during the subacute phase of cisplatin-induced testicular toxicity. Concomitant administration of methylsulfonylmethane (MSM) attenuated these biochemical and molecular alterations by reducing lipid peroxidation, restoring antioxidant enzyme activity, preserving intratesticular testosterone levels, and normalizing the cisplatin-induced GPX4 response. Collectively, these findings suggest that the observed effects of MSM observed during the subacute phase were more closely associated with preservation of redox homeostasis than with measurable changes in the inflammatory markers evaluated in the present study. Although biochemical alterations were clearly detectable, histopathological changes remained limited and Johnsen scores were not significantly different among the experimental groups. This may indicate that the present model represents an early or subclinical stage of testicular injury in which endogenous antioxidant defense mechanisms may have contributed to maintaining seminiferous tubular integrity despite measurable biochemical disturbances. However, because only a single subacute time point was evaluated, these findings should not be interpreted as evidence of a temporal sequence between biochemical and histopathological changes.

Oxidative stress is widely recognized as a central mechanism underlying cisplatin-induced testicular toxicity [1,6,17]. Excessive generation of reactive oxygen species (ROS) following cisplatin administration disrupts cellular redox balance, promotes lipid peroxidation, and impairs the endogenous antioxidant defense system, ultimately compromising testicular function [18,19]. Consistent with this concept, numerous experimental studies have demonstrated increased malondialdehyde (MDA) levels together with reduced activities of major antioxidant enzymes, including superoxide dismutase (SOD), catalase (CAT), and glutathione peroxidase (GPX), following cisplatin exposure [18,20,21]. This oxidative imbalance has been closely associated with impaired steroidogenesis and deterioration of testicular function [18,20,21]. Experimental studies evaluating different antioxidant compounds have likewise shown that attenuation of lipid peroxidation accompanied by restoration of endogenous antioxidant enzyme activity represents one of the principal mechanisms protecting against cisplatin-induced reproductive toxicity [4,18,22,23].

The findings of the present study closely parallel these previous observations. Cisplatin administration significantly increased testicular MDA levels while simultaneously reducing SOD, CAT, and GPX activities, indicating disruption of the antioxidant defense system [4,18,22,23]. In contrast, MSM administration markedly attenuated lipid peroxidation, restored SOD and CAT activities, and partially improved GPX activity, demonstrating a substantial recovery of testicular antioxidant capacity [16,22,24]. Notably, these biochemical improvements were accompanied by preservation of intratesticular testosterone levels despite the absence of marked inflammatory or histopathological alterations, suggesting that oxidative stress preceded overt structural injury in this subacute model [25,26]. Collectively, these findings indicate that cisplatin primarily disrupted testicular redox homeostasis, whereas MSM mitigated this imbalance by preserving endogenous antioxidant defense [12,16,25,26].

Oxidative stress is closely linked to impairment of Leydig cell function during cisplatin-induced testicular toxicity [27]. Excessive reactive oxygen species disrupt mitochondrial function and steroidogenic enzyme activity, resulting in reduced testosterone biosynthesis before extensive structural degeneration becomes evident [18,27]. Recent experimental studies have consistently reported decreased intratesticular testosterone following cisplatin exposure and have shown that antioxidant-based interventions preserve steroidogenic function by limiting oxidative damage [7,18,27]. Similar findings have been described for glycyrrhizin, morin hydrate, and other antioxidant compounds, in which restoration of redox homeostasis was associated with recovery of testosterone production [7,22,27]. In agreement with previous reports, cisplatin administration significantly reduced intratesticular testosterone concentrations, whereas MSM co-administration was associated with higher intratesticular testosterone levels. This observation was accompanied by improvements in antioxidant enzyme activities, particularly CAT [22]. However, because circulating reproductive hormones (LH and FSH) and additional steroidogenic markers were not evaluated, these findings should not be interpreted as direct evidence of preserved Leydig cell function or intact steroidogenic activity. Consistent with this observation, correlation analysis demonstrated a positive association between CAT activity and intratesticular testosterone concentrations. Nevertheless, correlation analysis alone cannot establish causal or mechanistic relationships; therefore, these findings may be regarded as complementary and exploratory.

GPX4 is a key phospholipid hydroperoxidase that protects cellular membranes against oxidative damage by reducing phospholipid hydroperoxides and limiting ferroptotic cell death [28]. Recent evidence has highlighted the importance of GPX4 as an early adaptive component of the antioxidant response during cisplatin-induced oxidative injury [8]. Experimental studies have shown that cisplatin-induced oxidative stress activates GPX4-associated defense pathways in several tissues, including the testis, where increased GPX4 expression has been interpreted as a compensatory response to excessive lipid peroxidation rather than a direct indicator of irreversible tissue damage [23,28]. Similarly, studies investigating ferroptosis-related pathways have suggested that activation of the GPX4 axis represents an early cellular attempt to preserve membrane integrity and maintain redox homeostasis under conditions of increased oxidative burden [8,29].

Our findings are consistent with previous reports describing GPX4 upregulation under oxidative stress conditions; however, the present study does not directly establish the underlying mechanism. Cisplatin administration significantly increased GPX4 protein expression despite the absence of marked histopathological injury, which may reflect a compensatory antioxidant response during the early phase of testicular toxicity. In contrast, co-administration of MSM restored GPX4 expression toward control levels in the present study. Considering that MSM simultaneously reduced lipid peroxidation and improved endogenous antioxidant enzyme activity, normalization of GPX4 expression may reflect attenuation of the oxidative burden rather than suppression of antioxidant defense itself. However, this interpretation requires confirmation through the evaluation of additional ferroptosis-related markers. GPX4 is widely recognized as a key regulator of ferroptosis because it protects membrane phospholipids from lipid peroxidation. Although the precise mechanisms cannot be established from the present study, these findings may be consistent with dynamic regulation of GPX4, which is dynamically regulated during the early stages of cisplatin-induced oxidative stress, rather than direct evidence of ferroptosis, and additional ferroptosis-related markers would be required to further clarify this relationship [6,8,23]. In contrast to GPX4, HO-1 protein expression showed only a modest upward trend following cisplatin administration, and no statistically significant differences were observed among the experimental groups. HO-1 is a stress-inducible cytoprotective enzyme that is activated by a wide range of oxidative and inflammatory stimuli through multiple signaling pathways [10,30]. Its induction is known to depend on the intensity and duration of cellular stress, and previous studies have demonstrated that HO-1 expression may exhibit tissue- and time-dependent regulation during oxidative injury [30,31,32]. Therefore, the absence of significant changes in HO-1 expression in the present study may be related to the relatively early subacute time point evaluated. In addition, the relatively moderate cisplatin dose (7 mg/kg) and the short observation period (10 days) may also have been insufficient to induce a robust HO-1 response. These findings indicate only that HO-1 protein expression was not significantly altered under the experimental conditions and sampling time examined and should not be interpreted as evidence for the absence of oxidative or inflammatory responses. Collectively, these findings suggest that GPX4 expression appeared to be more responsive than HO-1 under the experimental conditions and at the sampling time evaluated in the present study.

Although inflammation has been recognized as an important component of cisplatin-induced tissue injury, its contribution appears to depend on the severity and duration of oxidative stress. Previous experimental studies using prolonged treatment protocols or higher cumulative cisplatin doses have reported significant increases in pro-inflammatory cytokines, including IL-6 and TNF-α, together with extensive histopathological damage [7]. In contrast, studies evaluating earlier stages of cisplatin toxicity have suggested that oxidative stress may precede the development of a pronounced inflammatory response [7,27]. These discrepancies are likely related to differences in experimental design, including cisplatin dose, duration of exposure, sampling time, and tissue-specific responses. Consistent with this concept, neither IL-6 nor TNF-α levels differed significantly among the experimental groups in the present study despite clear evidence of oxidative imbalance. These findings indicate that the specific inflammatory markers evaluated were not significantly altered under the present experimental conditions and at the investigated subacute time point [6,22]. However, these results should not be interpreted as evidence for the absence of inflammatory processes, since other inflammatory mediators or different temporal responses were not assessed. Therefore, the present data primarily support the occurrence of early oxidative alterations, whereas the contribution of inflammatory mechanisms requires further investigation.

Histopathological alterations are generally regarded as a downstream consequence of sustained oxidative injury in cisplatin-induced testicular toxicity [27,33]. Previous experimental studies employing longer observation periods or repeated cisplatin administration have consistently reported marked degeneration of seminiferous tubules, disruption of spermatogenic organization, and significant reductions in Johnsen score [7,27,33]. In contrast, studies focusing on earlier stages of injury suggest that biochemical and molecular alterations may precede overt structural degeneration, reflecting the temporal progression of testicular damage rather than simultaneous development of all pathological changes [27,28,34]. Consistent with this concept, only mild histomorphological alterations were observed in the present study. Although focal tubular deformation, limited germ cell sloughing, and occasional vacuolization were detected following cisplatin administration, quantitative evaluation demonstrated no significant differences in Johnsen score among the experimental groups. Likewise, seminiferous tubular architecture remained largely preserved despite clear biochemical evidence of oxidative imbalance and impairment of antioxidant defense. These findings suggest that the subacute experimental model captured an early stage of testicular injury in which molecular and biochemical disturbances had already developed, whereas structural degeneration had not yet become prominent. Accordingly, the limited histopathological alterations observed in the present study should be interpreted in the context of the relatively short experimental period rather than as evidence against the presence of early testicular toxicity.

Although correlation analysis cannot establish causal relationships, it provides complementary information regarding the associations among the measured variables. The strongest positive correlations were observed between CAT activity and intratesticular testosterone concentrations, as well as between SOD and CAT, whereas CAT was negatively correlated with MDA. These associations were generally consistent with the overall biochemical findings but should be interpreted with caution, as they do not demonstrate direct biological interactions. Likewise, the positive correlation between IL-6 and SOD should be interpreted cautiously because IL-6 concentrations did not differ significantly among the experimental groups. Therefore, these findings should be considered exploratory and hypothesis-generating rather than evidence of causal relationships. Similar associations between antioxidant capacity and endocrine function have been reported in experimental studies evaluating oxidative testicular injury [7,27,28]. These observations are consistent with the present findings but should not be interpreted as evidence of a direct mechanistic relationship.

To further strengthen the translational potential of our study, the 500 mg/kg dose of MSM administered to rats can be converted to the human equivalent dose (HED) using the body surface area (BSA)-based interspecies dose conversion method recommended by the U.S. Food and Drug Administration (FDA). According to this method, the HED (mg/kg) is calculated by multiplying the animal dose (mg/kg) by the ratio of the species-specific Km (body surface area conversion) factor to the human Km factor [35,36].

Conversion of the animal dose to the human equivalent dose (HED) [35,36]:

HED (mg/kg) = Animal dose (mg/kg) × (Km (animal)/Km (human))

Using the FDA-recommended K_m_ values for rats (K_m_ = 6) and adult humans (K_m_ = 37), the 500 mg/kg dose of MSM employed in the present study corresponds to an HED of approximately 81 mg/kg, equivalent to approximately 4.9 g/day for a 60-kg adult. Nevertheless, this conversion should be interpreted solely as a theoretical estimate intended to facilitate translational interpretation, as it does not account for interspecies differences in pharmacokinetics, pharmacodynamics, bioavailability, metabolism, or safety profiles. Therefore, extrapolation of these findings to humans should be approached with caution, and the determination of a clinically relevant and safe dose will require comprehensive pharmacokinetic studies, toxicological evaluations, and well-designed clinical trials.

The present study has several limitations that should be considered when interpreting the findings. First, the experimental model was limited to a subacute period following a single cisplatin administration; therefore, the long-term effects of MSM on testicular function could not be evaluated. Second, reproductive outcomes such as sperm quality, fertility potential, and mating performance were not assessed. Furthermore, although intratesticular testosterone concentrations were determined, circulating testosterone, luteinizing hormone (LH), follicle-stimulating hormone (FSH), and additional steroidogenic markers were not evaluated. Therefore, definitive conclusions regarding Leydig cell function or steroidogenesis cannot be drawn from the present findings. In addition, although GPX4 and HO-1 protein expression were investigated, other ferroptosis-related markers were not evaluated. Therefore, the mechanistic interpretation of the observed changes in GPX4 expression should be made with caution, as additional ferroptosis-associated proteins and lipid peroxidation-related markers would be required to further clarify the underlying mechanisms. Future studies incorporating longer observation periods together with comprehensive reproductive, endocrine, and molecular analyses, including a broader panel of ferroptosis-related markers, may provide further insight into the mechanisms underlying the beneficial effects of MSM against cisplatin-induced testicular toxicity.

## 5. Conclusions

The findings of the present study indicate that subacute cisplatin administration was associated with disruption of testicular redox homeostasis, characterized by increased lipid peroxidation, impaired antioxidant defense, and reduced intratesticular testosterone concentrations before marked histopathological alterations became evident. MSM administration was associated with attenuation of these alterations through reducing oxidative stress, improving antioxidant enzyme activities, maintaining intratesticular testosterone concentrations, and restoring GPX4 expression toward control levels. Collectively, these findings suggest that the observed effects of MSM during the early phase of cisplatin-induced testicular toxicity may be more closely associated with the preservation of redox homeostasis than with measurable changes in the inflammatory markers evaluated in the present study. Nevertheless, additional studies incorporating longer observation periods, functional reproductive outcomes, and a broader panel of ferroptosis-related markers are warranted to further clarify the underlying mechanisms and the therapeutic potential of MSM in preserving male reproductive health during cisplatin chemotherapy.

## Figures and Tables

**Table 1:** Effects of methylsulfonylmethane (MSM) on oxidative stress biomarkers and antioxidant defense parameters in a subacute cisplatin-induced testicular toxicity model.

Measured Parameters	C	CIS	MSM	CIS + MSM	*p* Value
^1^ SOD (ng/g tissue)	84 ± 5.3	72 ± 3.5 ^a^*	84 ± 12 ^b^*	84 ± 11 ^b^*	0.035
^1^ GPX (ng/g tissue)	746 ± 65	637 ± 13 ^a^**	708 ± 65 ^b^*	678 ± 51 ^a^*	0.006
^1^ CAT (ng/g tissue)	1072 ± 71	866 ± 79 ^a^***	1013 ± 142 ^b^**	1021 ± 99 ^b^**	0.003
^2^ MDA (nmol/g tissue)	31 (27.7–33.1)	37 (36.1–38.7) ^a^*	33 (30.2–35.0) ^b^*	31 (29.9–34.2) ^b^*	0.025
^2^ GSH (mmol/g tissue)	4.54 (4.0–4.9)	3.88 (3.7–4.4)	4.01 (3.7–4.3)	4.06 (3.8–4.3)	0.434

*: *p* < 0.05, **: *p* < 0.01, ***: *p* < 0.001. a: Significantly different compared with the C group (SOD: *p* = 0.016; GPX: *p* = 0.001; CAT: *p* < 0.001; MDA: *p* = 0.018). b: Significantly different compared with the CIS group (SOD: MSM *p* = 0.016, CIS + MSM *p* = 0.015; GPX: *p* = 0.020; CAT: *p* = 0.007; MDA: MSM *p* = 0.010, CIS + MSM *p* = 0.018). ^1^ One-way ANOVA, LSD; ^2^ Kruskal–Wallis, Mann–Whitney U test.

**Table 2:** Effects of methylsulfonylmethane (MSM) on intratesticular testosterone levels in a subacute cisplatin-induced testicular toxicity model.

Measured Parameters	C	CIS	MSM	CIS + MSM	*p* Value
^1^ Testosterone (mIU/g tissue)	0.22 (0.21–0.23)	0.21 (0.17–0.21) ^a^*	0.20 (0.20–0.20) ^a^*	0.23 (0.21–0.23) ^b^*^c^*	0.013

*: *p* < 0.05. a: Significant compared with the Control group (CIS, *p* = 0.037; MSM, *p* = 0.018). b: Significant compared with the CIS group (CIS + MSM, *p* = 0.028). c: Significant compared with the MSM group (CIS + MSM, *p* = 0.011). ^1^ Kruskal–Wallis.

**Table 3:** Effects of methylsulfonylmethane (MSM) on intratesticular inflammatory cytokine levels in a subacute cisplatin-induced testicular toxicity model.

Measured Parameters	C	CIS	MSM	CIS + MSM	*p* Value
^1^ IL-6 (ng/g tissue)	0.141 ± 0.01	0.11 ± 0.02	0.12 ± 0.02	0.12 ± 0.04	0.114 *
^1^ TNF-α (ng/g tissue)	5 ± 0.47	4.41 ± 0.99	4.46 ± 0.91	4.19 ± 0.76	0.253 *

* *p* > 0.05. No statistically significant differences were observed among the experimental groups for IL-6 (*p* = 0.114) or TNF-α (*p* = 0.253). ^1^ One-way ANOVA followed by the LSD post hoc test.

**Table 4:** Descriptive statistics for Johnsen testicular biopsy scores (JTBS) across experimental groups.

**Group Name**	***n***	**Mean ± SD**		**Median (IQR)**		
Control *	7	9.886 ± 0.107		9.900 (0.200)		
Cisplatin	8	9.650 ± 0.316		9.750 (0.200)		
MSM	8	9.863 ± 0.130		9.900 (0.200)		
Cisplatin + MSM	7	9.686 ± 0.381		9.700 (0.400)		
**Kruskal–Wallis Analysis of Johnsen Testicular Biopsy Scores (JTBS) Across Experimental Groups**						
**Group**		**n**	**Mean Rank**	**Kruskal–Wallis H**	***df***	***p*-Value**
Control		7	19.71	5.745	3	0.125
Cisplatin		8	10.31			
MSM		8	18.44			
Cisplatin + MSM		7	13.86			

**Note.** Values are presented as mean ± standard deviation and median (interquartile range). * One animal in the control group died during the experimental period. In addition, one animal in the CIS + MSM group was found dead in its cage on the final experimental day before tissue collection. Consequently, histopathological analyses in the Control and CIS + MSM groups were performed using the available tissue samples. Missing values resulting from technical staining or tissue-processing issues were treated as missing data and were not replaced with zero. Analyses were performed using the available valid observations for each outcome. Between-group comparisons were performed using the Kruskal–Wallis test. Since the overall tests were not statistically significant, pairwise post hoc comparisons were not performed. *p* < 0.05 was considered statistically significant.

## Data Availability

The datasets generated and/or analyzed during the current study are available from the corresponding author on reasonable request.

## Abbreviations

The following abbreviations are used in this manuscript:

MSM	Methylsulfonylmethane
CIS	Cisplatin
GPX	Glutathione Peroxidase
GPX4	Glutathione Peroxidase 4
HO-1	Heme Oxygenase-1
IL-6	Interleukin-6
MDA	Malondialdehyde
TNF-α	Tumor Necrosis Factor-alpha
ROS	Reactive Oxygen Species
SOD	Superoxide Dismutase
H&E	Hematoxylin and Eosin
IHC	Immunohistochemistry
CAT	Catalase
WB	Western Blot
HED	Human Equivalent Dose
K_m_	Body surface area conversion factor
